# Adverse Events Associated with BNT162b2 and AZD1222 Vaccines in the Real World: Surveillance Report in a Single Italian Vaccine Center

**DOI:** 10.3390/jcm11051408

**Published:** 2022-03-04

**Authors:** Maria Costantino, Carmine Sellitto, Valeria Conti, Graziamaria Corbi, Francesco Marongiu, Giovanni Genovese, Giuseppina Moccia, Mario Capunzo, Anna Borrelli, Pasquale Pagliano, Mario Farroni, Grazia Maria Lombardi, Maria Giovanna Elberti, Amelia Filippelli, Francesco De Caro

**Affiliations:** 1 Department of Medicine, Surgery and Dentistry “Scuola Medica Salernitana”, University of Salerno, 84081 Baronissi, Italy; csellitto@unisa.it (C.S.); vconti@unisa.it (V.C.); gmoccia@unisa.it (G.M.); mcapunzo@unisa.it (M.C.); ppagliano@unisa.it (P.P.); m.farroni@studenti.unisa.it (M.F.); afilippelli@unisa.it (A.F.); fdecaro@unisa.it (F.D.C.); 2 Clinical Pharmacology Unit, University Hospital “San Giovanni di Dio e Ruggi d’Aragona”, 84121 Salerno, Italy; giovanni.genovese@sangiovannieruggi.it (G.G.); direzione.sanitaria@sangiovannieruggi.it (A.B.); grazia.lombardi@sangiovannieruggi.it (G.M.L.); maria.elberti@sangiovannieruggi.it (M.G.E.); 3 Department of Medicine and Health Sciences, University of Molise, 86100 Campobasso, Italy; graziamaria.corbi@unimol.it; 4 Italian Society of Gerontology and Geriatrics (SIGG), 50122 Florence, Italy; 5 DIIn, University of Salerno, 84084 Fisciano, Italy; fmarongiu@unisa.it

**Keywords:** COVID-19, vaccine, AEFI, BNT162b2, AZD1222, immunization

## Abstract

Aim: Despite huge efforts in developing specific drugs, vaccination represents the only effective strategy against COVID-19. Efficacy and safety of the COVID-19 vaccines were established during clinical trials. Nonetheless, it is very important to perform continuous surveillance. This observational study aimed to report potential Adverse Events Following Immunization (AEFI) following the first dose of two different COVID-19 vaccines, BNT162b2 and AZD1222. Methods and Results: Subjects who underwent vaccination at the vaccine center of the University Hospital of Salerno, Italy, were interviewed using an ad hoc questionnaire. AZD-vac group (*n* = 175) who received AZD1222 had a higher number of AEFI than the BNT-vac group (*n* = 1613) who received BNT162b2 (83% vs. 42%). The most frequent AEFI associated with AZD1222 and BNT162b2 were fever and pain at the injection site, respectively. The AZD-vac group used drugs to contrast AEFI more frequently than the BNT-vac group. In the BNT-vac group, there was a higher incidence of AEFI in women than in men (26.2% vs. 15.8%, *p* = 0.01), while no gender-related difference was observed in the AZD-vac group. Conclusions: AZD1222 and BNT162b2 vaccines show a good safety profile. Based on our results and literature data, there are no reasons to justify the reluctance that persists towards immunization.

## 1. Introduction

The COVID-19 pandemic has been the cause of more than 4.5 million deaths in the world. This event has changed citizens’ lifestyles [1] and amplified critical issues affecting the global health system [2]. The disease ranges from absence of symptoms to an acute respiratory distress syndrome (ARDS) requiring advanced life support [3]. Many drugs were immediately administered as “repurposed drugs”, including both antivirals such as favipiravir, lopinavir/ritonavir, umifenovir, ribavirin, and non-antiviral agents such as azithromycin, enoxaparin, baricitinib, tocilizumab (TCZ) [4,5]. Moreover, nonpharmacological treatments, including the *salus per aquam* (spa) therapy, whose efficacy is well-demonstrated [6,7,8,9,10,11,12], have been proposed as a complementary therapy to contrast several alterations associated with the so-called long-COVID syndrome.

Beyond other treatments, the vaccine represents the only effective therapeutic strategy available against COVID-19 up to date. The development of effective and safe vaccines against SARS-CoV-2 infection has been obtained thanks to a continuous dialogue between developers and scientific experts [13]. The research is still ongoing, and, currently, some vaccines are used worldwide, while others are still under investigation [14].

Vaccines have a history that started late in the 18th century that undoubtedly highlighted the importance of such a preventive approach [15]. Regarding the COVID-19 pandemic, in the absence of specific anti-COVID-19 drugs, only a global vaccine-related immunization allows for containing the spread of the virus, also reducing the occurrence of severe clinical cases and hospitalizations [16].

In Italy, the mRNA vaccine stated as BNT162b2 and the adenovirus vaccine named AZD1222 were largely administered. These molecules were developed by using different technologies, and they have peculiar characteristics with singular safety profiles [17,18]. Several factors, such as gender, might have influenced vaccine-associated clinical outcomes [19].

In EU countries, AZD1222 was dispensed mainly to adults less than 60 years old, while BNT162b2 has been more regularly administered in all age groups [20].

AZD1222 has been subject to a dispute due to a potential risk of thromboembolism, in particular of vaccine-induced immune thrombotic thrombocytopenia (VITT). Cases of immune thrombocytopenia and bleeding without thrombosis were also reported in subjects immunized with BNT162b2. VITT is characterized by a very low prevalence but is associated with rapid progression and a high mortality rate, requiring constant surveillance [21].

To date, in Italy, about 80% of the population completed the primary COVID-19 vaccination cycle. This percentage is rising, especially for an increased number of the first dose administration, following the introduction of new measures to contrast the spread of COVID-19, including mandatory vaccination for over 50-year-old people [22].

Nowadays, social restriction measures have been relaxed, and we are gradually going back to normal, thanks to the vaccines [23].

During clinical trials, the efficacy and safety of the vaccines were established. Nonetheless, it is very important to keep doing clinical trials and carry out continuous surveillance [24,25].

Indeed, because of the emergency, the clinical trials investigating the anti-SARS-CoV-2 vaccine effects had limited follow-up. Moreover, as in clinical trials performed for other drugs, some groups, especially frail people, children, and pregnant women, have not been adequately represented. To date, vaccination in Italy is taking place neatly and quickly, thanks to the capillary dissemination of hubs over the national territory. This is allowing to rapidly immunize the Italian citizens and to acquire new information on the safety profile of vaccines.

This study aimed to provide a surveillance report on BNT162b2 (BioNTech/Pfizer) and AZD1222 (AstraZeneca) vaccines from a single Italian center, reporting types of Adverse Events Following Immunization (AEFI), their severity and frequency, and the actions taken to solve them, also using gender and age-oriented analyses to explore the possible role of these factors in AEFI onset.

## 2. Materials and Methods

### 2.1. Study Design

In this observational study, subjects who underwent COVID-19 vaccination between 18 June and 2 August 2021, at the vaccine Center of the University Hospital of Salerno, Italy, were interviewed at the time of the second dose administration. They were split into two groups by the type of vaccines administered: BNT-vac group who received BNT162b2 (Pfizer/BioNTech) and AZD-vac group who received AZD1222 (Astra Zeneca). All participants gave their informed consent. The study obtained the approval of the Ethics Committee Campania Sud-Naples, Italy (N. 009850—7/2021).

### 2.2. Measurements

To record Adverse Events Following Immunization (AEFI), including the Serious ones (SAEFI), an ad hoc questionnaire was used. The enrolled subjects were asked to cross the box corresponding to adverse events already coded as AEFI (e.g., fever, pain at the injection site, etc.). To not lose important information, the subjects were requested to describe all other symptoms and their duration, as well as the actions taken to contrast such events. To classify the adverse events as “AEFI” or “SAEFI” the applicable Italian and European guidelines were used [17,26,27].

### 2.3. Statistical Analysis

A descriptive analysis of the general characteristics of the study population was performed. Conditions for the application of bivariate tests were assessed. For continuous variables, the results, expressed as mean ± standard deviation (SD), were analyzed with Student’s *t*-test for paired and unpaired normally distributed data. Categorical variables were summarized using frequencies and percentages, and to compare categorical variables between the groups, because of the data parametric distribution, we used the Chi-Square test (χ^2^), or the Fisher test when the variable included less than five subjects. A multivariate regression analysis was performed where appropriate. A *p*-value < 0.05 was considered statistically significant. The STATA 16 software package (STATA Corp., College Station, TX, USA) was used to perform all the analyses.

## 3. Results

A population of 1788 subjects was enrolled. All persons who had already received the first dose were gone to the center for the vaccine second dose administration. Nine of them switched from AZD to BNT because of changes in the statements of the Italian Regulatory Authority or because they had suffered from increased levels of D-dimer or of antibody title following the first dose of AZD. The BNT-vac group included 1613 subjects (45% men and 55% women) with a mean age of 64 ± 17.9 years (range: 12–99 years, Table 1). The AZD-vac group consisted of 175 subjects (51% men and 49% women), with a mean age of 46 ± 11.9 years (range: 23–69 years, Table 1). As shown in Table 1, the enrolled population was stratified for age decades [28]. The BNT-vac group was predominately (26.7%) constituted by subjects between 80 and 89-years old, while the AZD-vac group included more people (34.9%) between 50 and 59-year-old. Notably, several individuals could not receive AZD mainly because of their thrombotic risk (e.g., high D-dimer value, coagulation impairment, etc.) and age restriction (initially individuals aged ≥18 years older, and later aged ≥60 years older were eligible), according to the recommendations in force in Italy when this study was carried out.

All enrolled subjects were Caucasians. Two hundred and seventy-four (28%) subjects in the BNT-vac group and 55 (31%) in the AZD-vac group self-reported food and drugs allergy. At baseline, between the groups, differences regarding the age and the prevalence of food and drugs allergy, but not concerning gender were found (Table 1).

### 3.1. Incidence, Description, and Mean Duration of AEFI after Administration of BNT162b2 and AZD1222 First Dose

**Figure 1:**
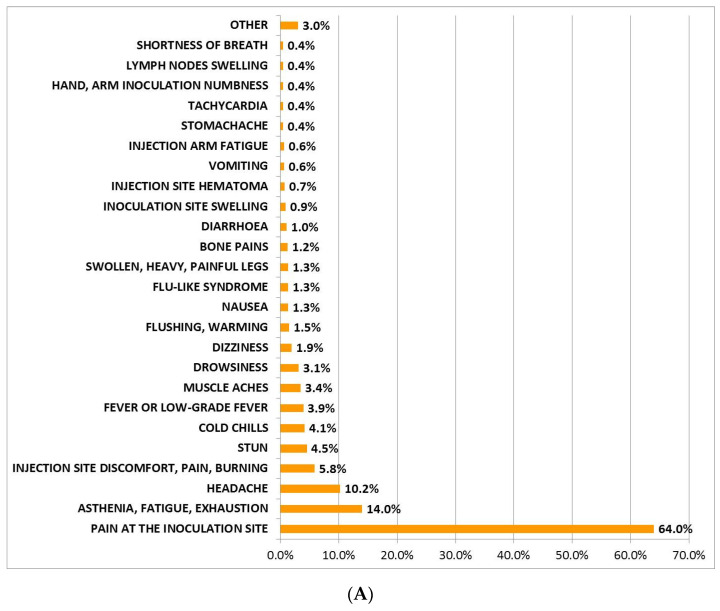

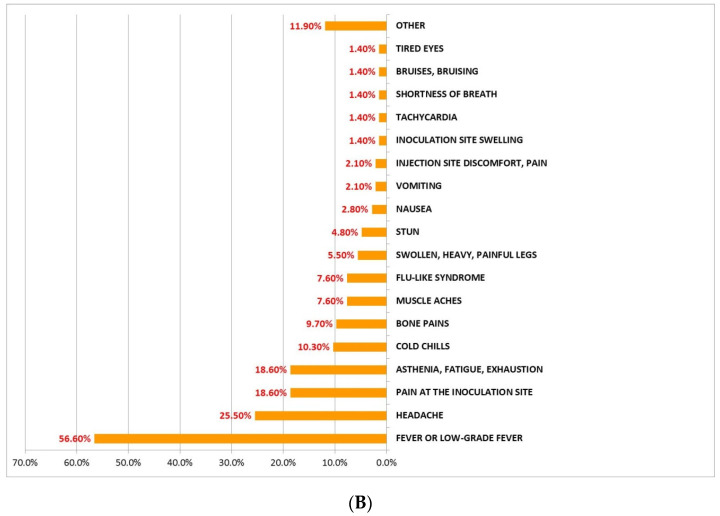
Incidence of AEFI reported in the BNT-vac group (**A**) and in the AZD-vac group (**B**). The category “Other” included symptoms with an incidence less than 0.4% in (**A**) and less than 1.4% in (**B**).

**Figure 2:**
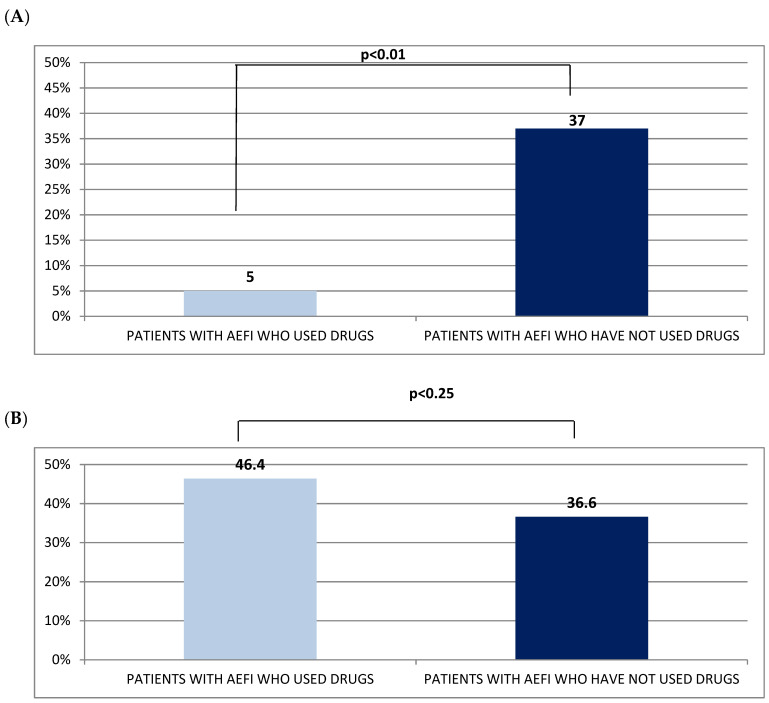
(**A**) Percentage of subjects in BNT-vac group (*n* = 684, 42%) who took drugs following the onset of AEFI after the administration of the first dose of vaccine; (**B**) Percentage of subjects in AZD-vac group (*n* = 145, 83%) who took drugs after the onset of AEFI following the first dose of vaccine.

Six hundred and eighty-four subjects (42%, mean age 58.5 ± 17.3 years, range: 12–94 years, 15.8% men and 26.2% women) who had received the first dose of BNT162b2 vaccine developed AEFI (Table 2). Figure 1A reports such AEFI and their incidence. In this figure, the category “Other” included symptoms with an incidence equal to 0.1% (stomatitis, hyper-salivation, bitter taste, tired eyes, peripheral cyanosis, plantar paresthesia, left chest pain, urticaria, hypotension, cold, injection site erythema, paresthesia at inoculation site, constipation) and 0.3% (sweating, reddened eyes, back pain, tingling, remarkable hungry). Among the subjects of the BNT-vac group who experienced AEFI (5%), some drugs were used (Figure 2A), mostly non-steroidal anti-inflammatory drugs (NSAIDs), in particular paracetamol. On average, the AEFI duration lasted 32.6 h ± 34.6 (range 0.25–504 h, Table 2).

One hundred and forty-five (83%, mean age of 45.0 ± 11.9, range: 23–69 years, 42% men and 41% women) subjects who had received the first dose of AZD1222 vaccine developed AEFI (Table 3). The AEFI type and incidence are described in Figure 1B. The category “Other” included symptoms with an incidence equal to 0.7% (swelling lip, sensation of closed throat, skin rash, numbness, cold lower limbs, chest constriction, photophobia, visual disturbances, diarrhea, stomatitis, back pain, remarkable hungry, dizziness, drowsiness, stomachache, weakness at inoculation site).

Notably, 81 subjects (46.4%) belonging to the AZD-vac group, presenting AEFI following the first dose of vaccine, used drugs (Figure 2B), in particular paracetamol or other NSAIDs (Table 3). On average, the AEFI duration was 37.1 h ± 90.6 (range 0.5–1080 h, Table 3).

### 3.2. AEFI after Administration of BNT162B2 or AZD1222 Based on Age

**Figure 3:**
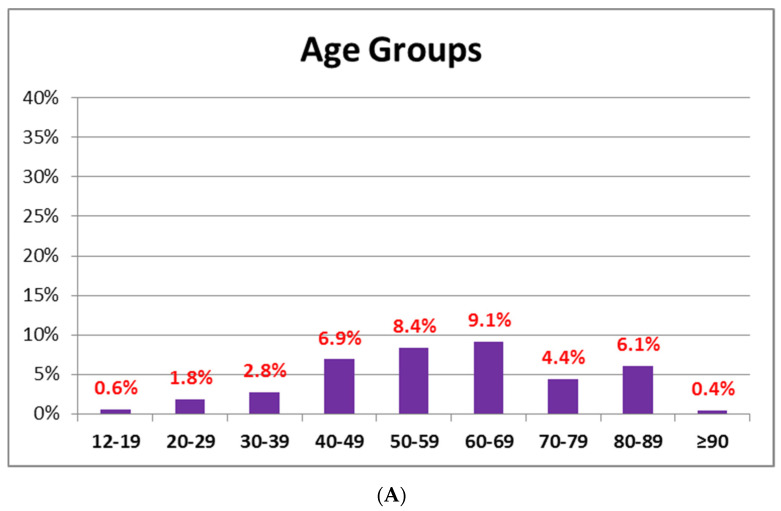

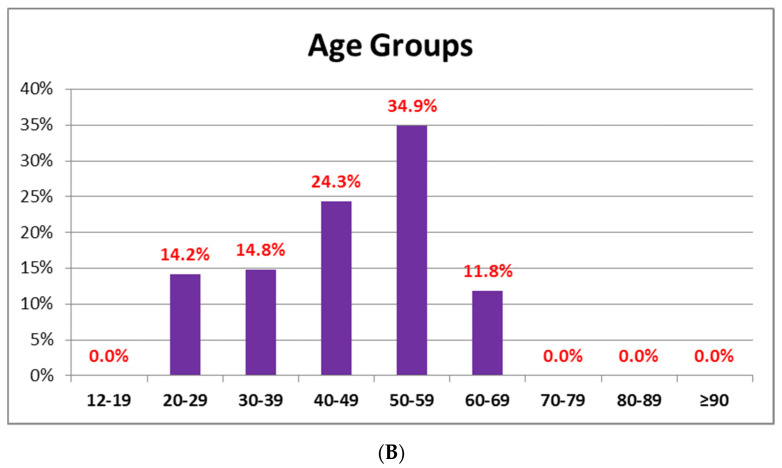
Percentage of AEFI in the vaccinated subjects stratified by age: 42% of the BNT-vac group (**A**); 83% of the AZD-vac group (**B**).

In the BNT-vac group, 42% of the subjects showed AEFI after the vaccine first dose administration. Most of these subjects (9%) belonged to the 60–69-year-old group, while the ≥90-years-old group (0.4%) was the least represented (Figure 3A). In the AZD-vac group, 83% of the subjects showed AEFI after the first dose administration: the most represented (34.9%) were the subjects included in the 50–59-years-old group, while the lowest (11.8%) belonged to the 60–69-years-old. None of the subjects belonging to the ≥90-years-old, 80–89-years-old, 70–79-years-old groups, as well as the 12–19-years-old group reported AEFI (Figure 3B).

### 3.3. AEFI after Administration of BNT162b2 or AZD1222 Based on Gender

**Figure 4:**
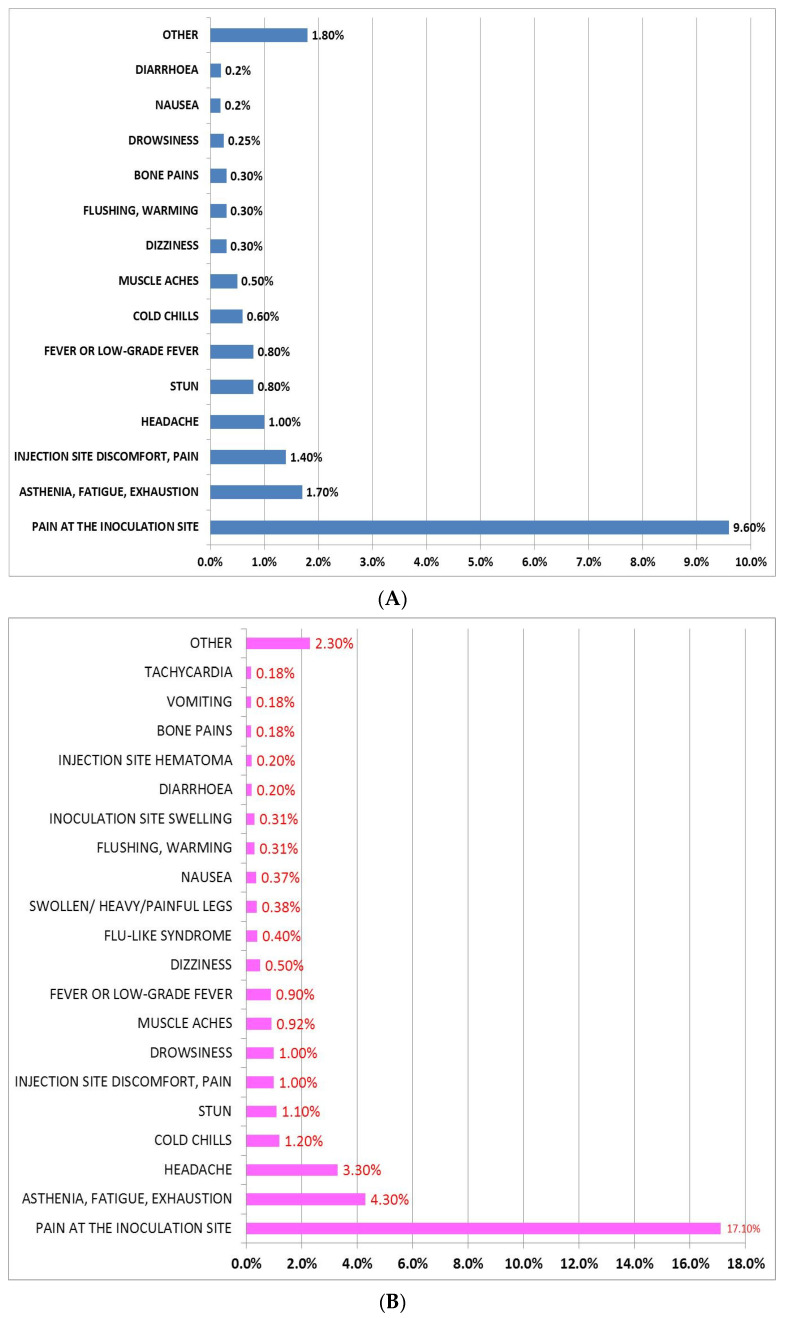
Incidence of the most frequent AEFI reported in men (**A**) and women (**B**) following the first dose of BNT162b2 vaccine. The category “Other” included the AEFI with an incidence less than 0.2% in (**A**) and less than 0.18% in (**B**).

A statistically significant (*p* = 0.01) higher prevalence of women (*n* = 428, 26.2%) was observed in the interviewed subjects who presented AEFI following the first dose administration of BNT162b2 (BNT-vac group) (Table 2). Furthermore, as shown in Table 2, stratifying by gender, no difference was found in mean age and age range. A higher duration (33.9 ± 37.8 h, *p* = 0.107) of AEFI was observed in women, without reaching statistical significance. Moreover, a greater use of drugs following AEFI after the BNT162b2 first dose administration was reported in women (3.6%) compared to men (1.4%, Table 2). The most used drug in both sexes was paracetamol with an incidence of 2.9% in women and 1.2% in men. Figure 4 shows the description and incidence of AEFI occurred in 15.8% of men and in 26.2% of women who experienced AEFI after the first dose administration of BNT162b2. In both sexes, the AEFI with the highest incidence was pain at the inoculation site (9.6% in men and 17.1% in women), followed by asthenia, fatigue, and exhaustion with an incidence of 1.7% in men and 4.3% in women (Figure 4). The men belonging to the 50–59-year-old group (24.4%) showed the highest incidence of AEFI, while the 60–69-year-old group (24%) was the most affected among women, followed by the 60–69-year-old group (20.4%) for men and 50–59-year-old group (18.6%) for women. Moreover, among men, we found the following percentage: 17.6% in the 40–49, 13.6% in the 70–79, 13.2% in the 80–89, 4.8% in the 30–39, 4% in the 20–29, 1.2% in the 12–19 and 0.8% in the ≥90 years-old groups. Among women, we observed 16.6% in the 40–49, 16.1% in the 80–89, and 9.3% in the 70–79, 8.3% in the 30–39, 4.6% in the 20–29, 1.5% in the 12–19 and 1% in the ≥90 years old groups. No statistically significant gender differences were observed in the interviewed subjects who presented AEFI after the first dose administration of AZD1222 (*n* = 145, 83% of total interviewed) (Table 3).

**Figure 5:**
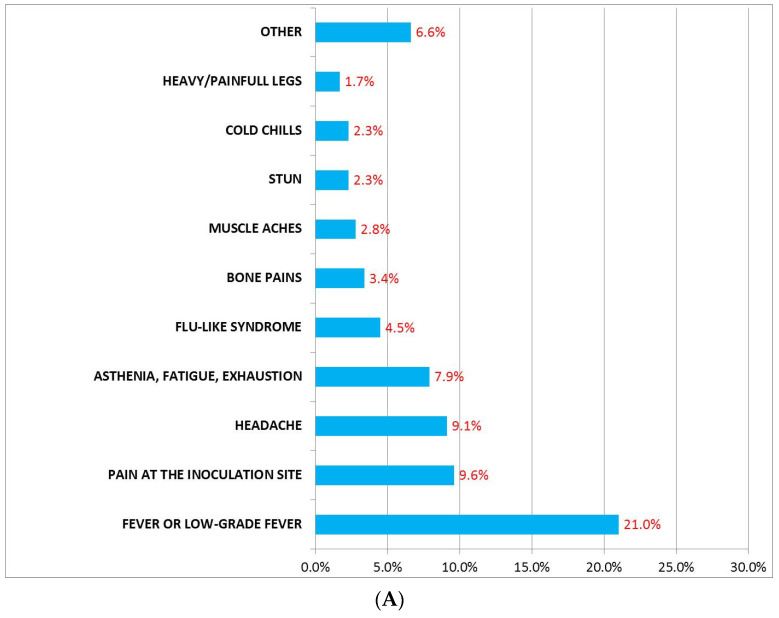

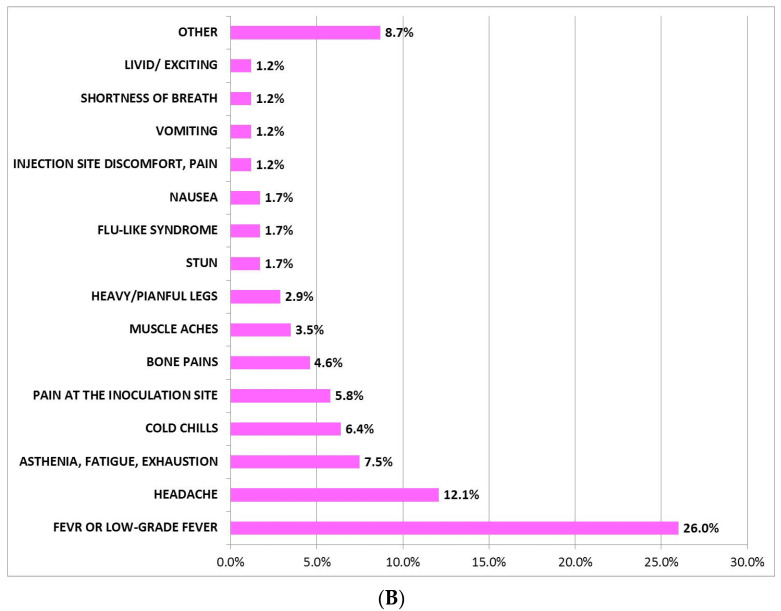
Incidence of the most frequent AEFI reported by men (**A**) and women (**B**) following the first dose of AZD1222 vaccine. The category “Other” included the AEFI with an incidence less than 1.7% in (**A**) and less than 1.2% in (**B**).

As shown in Table 3, there were no statistically significant differences (*p* = 0.697) in AEFI incidence and duration stratifying population by gender, while there was a larger use (*p* = 0.041) of drugs following the onset of AEFI in women (26.4%) compared to men (20%). In the AZD1222 group, the most used drug was again paracetamol with an incidence of 23.5% in women and 18.2% in men, without a statistically significant difference between the sexes (Table 3). Figure 5 shows the type and incidence of AEFI detected in 42% of men and 41% of women who experienced AEFI after the first dose administration of AZD1222. In both sexes, the AEFI with the highest incidence was low-grade fever (21% in men and 26% in women), followed by headache (12.1%) in women and pain at the inoculation site (9.6%) in men (Figure 5). Men belonging to the 50–59-year-old group (35.1%) showed the highest incidence of AEFI, while the 40–49-year-old and 50–59-year-old groups (25.3%) were the most affected among women. This rate was followed by that observed in the 40–49-year-old group (29.7%) among men and the 30–39-year-old group (24%) among women. The men belonging to the 20–29-year-old group showed 13.5%, the 30–39 and 60–69-year-old groups 10.8% incidence of AEFI, while no AEFI were reported among the other age groups. Among the women, we highlighted 17% incidence of AEFI in the 20–29-year-old group, 8.4% in the 60–69-year-old group, and no AEFI in the other age groups.

### 3.4. Differences of AEFI between BNT162b2 and AZD1222

We found that the AZD group experienced more frequently than the BNT group symptoms such as fever (46.8%, *p* < 0.0001), headache (21.1%, *p* < 0.0001), asthenia, fatigue, exhaustion (15.4%, *p* < 0.0001), chills (8.6%, *p* < 0.0001), bone pain (8%, *p* < 0.0001) (Table S1). In turn, the BNT group showed a significantly higher incidence of pain at the injection (26.9%, *p* = 0.001) than the AZD group. Moreover, performing a multivariate logistic regression, with the group as the dependent variable and as independent factors the gender, the age, and the variables that resulted significant at the univariate analysis, we found that the best predictors to be undergone the AZD vaccination were to be men (OR 1.86, 95% CI 1.20 to 2.88; *p* = 0.005) and to have experienced, as AEFI, the fever (OR 28.94, 95% CI 15.94 to 52.56; *p* < 0.0001), headache (OR 4.92, 95% CI 2.70 to 8.98; *p* < 0.0001), swollen heavy painful legs (OR 10.12; 95% CI 2.84 to 36.07; *p* < 0.0001), and a flu-like syndrome (OR 6.91, 95% CI 2.06 to 23.19; *p* = 0.002). Conversely, the BNT vaccination was predicted by younger age (OR 0.96, 95% CI 0.95 to 0.97; *p* < 0.0001) and the pain at the injection site as AEFI (OR 0.42, 95% CI 0.25 to 0.74; *p* = 0.002; Table S2).

## 4. Discussion

For COVID-19 vaccines, as well as for other treatments used during the pandemic, there is the need to accumulate as much data as possible. Indeed, the response to anti-COVID-19 drugs, often used according to the concept of “drug repurposing”, has been variable and dependent on the patients’ clinical setting [4,5]. For example, remdesivir, an adenosine analog inhibiting viral RNA-dependent-RNA-polymerase, is now part of the COVID-19 treatment protocols, but its efficacy and safety are still under-studied, even if it has been used for COVID-19 patients since the early months of the pandemic [29].

A complete vaccination cycle represents the only effective strategy to avoid worsened outcomes related to the COVID-19 syndrome, including hospitalization and admission to intensive care units also in vulnerable patients [30,31,32]. Furthermore, it has been repeatedly underlined the importance to expand, as soon as possible, the audience of the vaccinated subjects [33].

However, as for every drug, even vaccines can be characterized by differences in efficacy and safety.

In the present study, we reported AEFI that occurred following the administration of the two vaccines, mainly used during the first wave of the COVID-19 pandemic.

According to the Italian Regulatory Agency, AEFI mainly happened in people with an average age of 49 years following the inoculation of BNT162b2-BioNTech/Pfizer, mRNA-1273-Moderna, AZD1222-AstraZeneca, and COVID-19 Janssen Vaccine -Janssen Cilag [34]. These adverse events occurred in different organs/systems with a frequency of about 90% after the first dose administration. SAEFI associated with BNT162b2 amounted to 9.6% versus 17% associated with AZD1222 [24].

Following inoculation of the vaccine mRNA-1273-Moderna, a rate of 0.95 myocarditis/myopericarditis cases per 100,000 vaccine recipients was reported [35].

A multicenter cohort study, conducted in the UK, described 70 cases of cerebral venous thrombosis associated with vaccine-induced immune thrombotic thrombocytopenia (VITT) that occurred after the first dose of AZD1222. However, the authors concluded that VITT was a rare side-effect, whose risk was outweighed by the benefit of immunization against COVID-19 [36].

Our report showed that, despite the AZD-vac group including subjects significantly younger than those in the BNT-vac group, the AEFI were more often reported in the first group than in the second one (83% vs. 42%).

Patients who experienced AEFI in the AZD-vac group recurred more frequently to pharmacological therapy compared to those included in the BNT-vac group (46.4% vs. 5%). In both groups, the most used drugs were NSAIDs, in particular paracetamol (41.7% in AZD-group vs. 4.14% in BNT-group). Moreover, stratifying by gender, the use of paracetamol was greater in women compared to men, regardless of the vaccine type. However, such gender difference was statistically significant only for the BNT162b2 vaccine.

Regarding BNT162b2, the most reported AEFI were the pain at the injection site followed by mild symptoms such as asthenia, fatigue, and headache. On the contrary, the AEFI most frequently reported by the subjects vaccinated with AZD1222 were fever and headache.

Stratifying the population by age, the subjects the majority of AEFI were 50–59-year-old and 60–69-year-old in the AZD-group and the BNT-group, respectively.

Notably, we found a higher incidence of AEFI in women than in men in the BNT-group, while no difference by gender was found in the AZD-group (41% women vs. 42% men). Moreover, the women in the AZD-group were younger than the ones in the BNT-group. Among the women of the AZD-group, the most affected were included in the age range of 40–46 years and 50–59-years. Regardless of vaccine type, the men including in the 50–59-year-old group were the most affected (35.14% in the AZD group and 24.4% in the BNT-group). Hence, the incidence of AEFI associated with both the first dose of the analyzed vaccines can be dependent on the age of the subjects that can influence the immune system reactivity [37,38].

As already discussed, and in agreement with the literature [39,40], our study showed that AEFI onset can be influenced by gender. This could be related to the opposite role of estrogens and testosterone. Estrogens stimulate the immune system to produce a great number of antibodies, as demonstrated in response to the flu vaccine, while testosterone has an immunosuppressive action [39]. There is also a possible influence exerted by genetic factors. In this regard, it is interesting to underline that several genes regulating the immune system are located on the X chromosome. It has been reported that 15% of genes are expressed at higher levels in women than in men because of escape from X inactivation. This could concur also to explain why some autoimmune diseases are more common in women [40].

Moreover, pharmacokinetic parameters, mainly drug metabolism, differ between the two sexes. Regrettably, no lower doses of the COVID-19 vaccines have been tested in women to prevent a possible excessive immune reactivity. Further evidence is needed to explain these findings and hypotheses.

A limitation of the present study includes the difference in the sample size of the two vaccinated patient groups. However, this reflects the greater availability of the BNT162b2 at the vaccination center according to the statement of the Regulatory Agencies, and to the later introduction of AZD1222 in the clinical practice. Another concern regards the study design (monocentric) that does not allow for a generalization of the obtained results.

Moreover, the self-reporting method adopted in the study could lead to over or under-estimate the adverse events. On the other hand, it represents a useful approach to expand the knowledge on COVID-19 vaccines safety.

## 5. Conclusions

This study highlighted some differences in the incidence and type of AEFI associated with BNT162b2 and AZD1222. However, both vaccines showed a favorable safety profile.

Based on our results and the literature, there is no reason to avoid COVID-19 vaccination. Mainly in this period in which countries, such as Italy, have stated that patients over 50 years of age must be vaccinated and many people are approaching the first dose of vaccine, there is a need to disseminate surveillance data to contrast hesitancy and reluctance of “no-vax people” towards the COVID-19 vaccination.

## Figures and Tables

**Table 1:** Main characteristics of patients belonging to the BNT-vac and AZD-vac groups.

	Total *n* = 1.788	BNT-Vac Group *n* = 1.613	AZD-Vac Group *n* = 175	*p* Value
Age, years				
mean ± SD	62.5 ± 18.2	64 ± 17.9	46 ± 11.9	0.001
median [range]	63 [12–99]	41 [12–99]	49 [23–69]	
GENDER, *n* (%)				
Men	817 (46)	728 (45)	89 (51)	0.39
Women	971 (54)	885 (55)	86 (49)	0.425
Self-reported Allergy to food or drugs, *n* (%)	329 (18)	274 (28)	55 (31)	0.001
Distribution by age, *n* (%)				
12–19	16 (0.9)	16 (1)	0 (0)	0.188
20–29	78 (4.4)	54 (3.4)	24 (14.2)	0.001
30–39	99 (5.5)	74 (4.7)	25 (14.8)	0.001
40–49	227 (12.7)	186 (11.9)	41 (24.3)	0.001
50–59	325 (18.2)	266 (17.0)	59 (34.9)	0.001
60–69	332 (18.6)	312 (19.9)	20 (11.8)	0.03
70–79	200 (11.2)	200 (12.8)	0 (0)	0.001
80–89	419 (23.4)	419 (26.7)	0 (0)	0.001
≥90	40 (2.2)	40 (2.6)	0 (0)	0.038

**Table 2:** Main characteristics stratified by gender evaluated in 42% of the interviewed subjects who presented AEFI following the first dose of BNT162b2 vaccine.

	Total *n* = 684	Men	Women	*p*
Age, years				
mean ± SD	58.5 ± 17.3	59.0 ± 16	58.0 ± 18	0.464
median [range]	59 [12–94]	59 [16–92]	60 [12–94]	
AEFI, *n* (%)	684 (42)	256 (15.8)	428 (26.2)	0.01
AEFI duration (hours)				
mean ± SD	32.6 ± 34.6	29.6 ± 25.3	33.9 ± 37.8	0.107
Range in hours	0.25–504	0.25–168	0.5–504	
N subjects with drugs after AEFI, *n* (%)	83 (12.1)	23 (1.4)	60 (3.6)	0.01
Drugs after AEFI, *n* (%)				
Paracetamol	68 (4.14)	19 (1.20)	49 (2.94)	0.01
Nimesulide	4 (0.22)	2 (0.10)	2 (0.12)	0.96
Ketoprofen	4 (0.22)	2 (0,10)	2 (0.12)	0.96
ASA	1 (0.06)	0 (0)	1 (0.06)	0.30
Diosmin	1 (0.06)	0 (0)	1 (0.06)	0.30
Lichtena cream	1 (0.06)	0 (0)	1 (0.06)	0.30
Disinfectants + vitamins	1 (0.06)	0 (0)	1 (0.06)	0.30
Paracetamol + ibuprofen	1 (0.06)	0 (0)	1 (0.06)	0.30
Paracetamol + ketoprofen	1 (0.06)	0 (0)	1 (0.06)	0.30
alcohol compressed	1 (0.06)	0 (0)	1 (0.06)	0.30

ASA, acetylsalicylic acid.

**Table 3:** Main characteristics by gender valuated in 83% of the interviewed subjects who presented AEFI following the first dose of AZD1222.

	Total	Men	Women	*p*
Age, years				
mean ± SD	45.0 ± 11.9	46.5 ± 11.6	43.3 ± 11.7	0.100
median [range]	47 [23–69]	48 [26–67]	45 [23–69]	
AEFI, *n* (%)	145 (83)	74 (42.0)	71 (41.0)	0.697
AEFI duration (hours)				
mean ± SD	37.1 ± 90.6	30.0 ± 23.3	44.3 ± 127.1	0.343
Range in hours	0.5–1080	0.5–168	0.5–1080	
N subjects with drugs after AEFI, *n* (%)	81 (46.4)	35 (20.0)	46 (26.4)	0.041
Drugs after AEFI, *n* (%)				
Paracetamol	73 (41.7)	32 (18.2)	41 (23.5)	0.07
Ketoprofen	1 (0.06)	1 (0.6)	0 (0	0.20
ASA	1 (0.06)	1 (0.6)	0 (0)	0.20
Nimesulide+ASA	1 (0.57)	0 (0)	1 (0.57)	0.20
Ibuprofen	2 (1.17)	1 (0.6)	1 (0.57)	0.97
Bilastine	1 (0.57)	0 (0)	1 (0.57)	0.21
Indomethacin + caffeine + Prochlorperazine	1 (0.57)	0 (0)	1 (0.57)	0.21
Paracetamol + ibuprofen + metoclopramide	1 (0.57)	0 (0)	1 (0.57)	0.21

ASA, acetylsalicylic acid.

## Data Availability

Not applicable.

## Supplementary Materials

The following supporting information can be downloaded at: https://www.mdpi.com/article/10.3390/jcm11051408/s1, Table S1: Differences in the incidence of Symptoms reported in AZD and BNT groups; Table S2: Logistic regression.
