# Supplementary material for: Adverse Events Associated with BNT162b2 and AZD1222 Vaccines in the Real World: Surveillance Report in a Single Italian Vaccine Center
Source: J Clin Med. 2022 Mar 4;11(5):1408. doi: 10.3390/jcm11051408 (PMC8911524; doi:10.3390/jcm11051408)
Supplement: Supplementary file 1 [file jcm-11-01408-s001.zip › jcm-1602981-supplementary.pdf]

Table S1: Differences in the incidence of Symptoms reported in AZD and BNT groups

	Total	AZD	BNT	p
ASTHENIA, FATIGUE, EXHAUSTION, n (%)	125 (6.99)	27 (15.43)	98 (6.08)	**<0.0001**
BACK PAIN, n (%)	3 (0.17)	1 (0.57)	2 (0.12)	0.170
BELLYACHE, n (%)	4 (0.22)	1 (0.57)	3 (0.19)	0.305
BITTER TASTE, n (%)	1 (0.06)	0 (0.00)	1 (0.06)	0.742
BONE PAIN, n (%)	22 (1.23)	14 (8.00)	8 (0.50)	**<0.0001**
BRUISES, ECCHYMOSIS, n (%)	2 (0.11)	2 (1.14)	0 (0.00)	**<0.0001**
CHEST CONSTRICTION, n (%)	1 (0.06)	1 (0.57)	0 (0.00)	**0.002**
CHILLS, n (%)	43 (2.40)	15 (8.57)	28 (1.74)	**<0.0001**
COLD LOWER LIMBS, n (%)	1 (0.06)	1 (0.57)	0 (0.00)	**0.002**
COLD, n (%)	1 (0.06)	0 (0.00)	1 (0.06)	0.742
CONSTIPATION, n (%)	1 (0.06)	0 (0)	1 (0.06)	0.742
COUGH, n (%)	1 (0.06)	0 (0.00)	1 (0.06)	0.742
DIARRHOEA, n (%)	8 (0.45)	1 (0.57)	7 (0.43)	0.796
DIZZINESS, n (%)	14 (0.78)	1 (0.57)	13 (0.81)	0.738
DROWSINESS, n (%)	22 (1.23)	1 (0.57)	21 (1.30)	0.405
FEELING OF CLOSED THROAT, n (%)	1 (0.06)	1 (0.57)	0 (0.00)	**0.002**
FEVER, n (%)	109 (6.10)	82 (46.86)	27 (1.67)	**<0.0001**
FLU LIKE SYNDROME, n (%)	20 (1.12)	11 (6.29)	9 (0.56)	**<0.0001**
FLUSHING WARMING, n (%)	10 (0.56)	0 (0.00)	10 (0.62)	0.296
HAND ARM INOCULATION NUMBNESS, n (%)	3 (0.17)	0 (0.00)	3 (0.19)	0.568
HEADACHE, n (%)	107 (5.98)	37 (21.14)	70 (4.34)	**<0.0001**
HYPERSALIVATION, n (%)	1 (0.06)	0 (0.00)	1 (0.06)	0.742
HYPOTENSION. n (%)	1 (0.06)	0 (0.00)	1 (0.06)	0.742
INJECTION ARM FATIGUE, n (%)	5 (0.28)	1 (0.57)	4 (0.25)	0.442
INJECTION SITE DISCOMFORT, n (%)	43 (2.40)	3 (1.71)	40 (2.48)	0.530
INJECTION SITE ERYTHEMA, n (%)	1 (0.06)	0 (0.00)	1 (0.06)	0.742
INJECTION SITE HEMATOMA, n (%)	5 (0.28)	0 (0.00)	5 (0.31)	0.461
INJECTION SITE SWELLING, n (%)	8 (0.45)	2 (1.14)	6 (0.37)	0.147
INOCULATION ARM PARESTHESIA, n (%)	1 (0.06)	0 (0.00)	1 (0.06)	0.742
LEFT CHEST PAIN, n (%)	1 (0.06)	0 (0.00)	1 (0.06)	0.742
LYNPHONODE SWELLING, n (%)	3 (0.17)	0 (0.00)	3 (0.19)	0.568
MUSCLE PAIN, n (%)	34 (1.90)	11 (6.29)	23 (1.43)	**<0.0001**
NAUSEA, n (%)	13 (0.73)	4 (2.29)	9 (0.56)	**0.011**
NUMBNESS, n (%)	1 (0.06)	1 (0.57)	0 (0.00)	**0.002**
PAIN INOCULATION SITE, n (%)	462 (25.84)	27 (15.43)	435 (26.97)	**0.001**
PERIPHERAL CYANOSIS, n (%)	1 (0.06)	0 (0.00)	1 (0.06)	0.742
PHOTOPHOBIA, n (%)	1 (0.06)	0 (0.00)	1 (0.06)	0.742
PLANTAR PARAESTHESIA, n (%)	1 (0.06)	0 (0.00)	1 (0.06)	0.742
REDDENED EYE, n (%)	2 (0.11)	0 (0.00)	2 (0.12)	0.641
REMARKABLE HUNGRY, n (%)	3 (0.17)	1 (0.57)	2 (0.12)	0.170
SHORTNESS OF BREATH, n (%)	5 (0.28)	2 (1.14)	3 (0.19)	**0.023**
SICKNESS, n (%)	2 (0.11)	1 (0.57)	1 (0.06)	0.056
SKIN RASH, n (%)	1 (0.06)	1 (0.57)	0 (0.00)	**0.002**
STOMATITIS, n (%)	2 (0.11)	1 (0.57)	1 (0.06)	0.056
STUN, n (%)	38 (2.13)	7 (4.00)	31 (1.92)	0.070
SWEATING, n (%)	2 (0.11)	0 (0.00)	2 (0.12)	0.641
SWELLING LIPS, n (%)	1 (0.06)	1 (0.57)	0 (0.00)	**0.002**
SWOLLEN HEAVY PAINFUL LEGS, n (%)	14 (0.78)	8 (4.57)	6 (0.37)	**<0.0001**
SWOLLEN LEGS, n (%)	3 (0.17)	0 (0.00)	3 (0.19)	0.568
TACHYCARDIA, n (%)	5 (0.28)	2 (1.14)	3 (0.19)	**0.023**
TINGLING, n (%)	3 (0.17)	0 (0.00)	3 (0.19)	0.568
TIRED EYES, n (%)	2 (0.11)	1 (0.57)	1 (0.06)	0.056
URTICARIA, n (%)	1 (0.06)	0 (0.00)	1 (0.06)	0.742
VISUAL DISTURBANCES, n (%)	1 (0.06)	0 (0.00)	1 (0.06)	0.742
VOMITING, n (%)	7 (0.39)	3 (1.71)	4 (0.25)	**0.003**

Table S2: Logistic regression. To determine the best predictors of the vaccination type, a logistic regression was performed by using as dependent variable the Group and as independent the variables that were significant at univariate analysis.

Group	Odds Ratio	Std. Err.	z	P>z	[95% Conf. Interval]	
GENDER (Male)	1.861676	0.4136595	2.80	0.005	1.204397	2.877656
AGE years	0.9582351	0.0060177	-6.79	0.000	0.9465128	0.9701025
ASTHENIA, FATIGUE, EXHAUSTION	1.845978	0.6002083	1.89	0.059	0.976031	3.491318
BONE_PAIN	3.452661	2.30574	1.86	0.064	0.9326282	12.78202
CHILLS	1.253267	0.6877045	0.41	0.681	0.4275275	3.673863
FEVER	28.94352	8.810064	11.06	0.000	15.9388	52.55899
FLU_LIKE_SYNDROME	6.907558	4.268413	3.13	0.002	2.057493	23.19053
HEADACHE	4.924153	1.509749	5.20	0.000	2.69993	8.980709
MUSCLE_PAIN	0.9562886	0.6055338	-0.07	0.944	0.2764379	3.308113
NAUSEA	4.293	4.343325	1.44	0.150	0.5909911	31.18464
PAIN_INOCULATION_SITE	0.4276371	0.1192237	-3.05	0.002	0.2476072	0.7385628
SHORTNESS_OF_BREATH	4.428591	5.339752	1.23	0.217	0.4168054	47.05413
SWOLLEN_HEAVY_PAINFU~S	10.11546	6.561416	3.57	0.000	2.836954	36.06778
TACHYCARDIA	0.272131	0.3850307	-0.92	0.358	0.0169995	4.356322
VOMITING	4.072253	5.048986	1.13	0.257	0.3584857	46.25915

Group: 0= BNT, 1 =AZD.
